# Prevalence and Biochemical Associations of Fever in Adults With Reverse Transcription Polymerase Chain Reaction Proven Coronavirus Disease Presenting at Tertiary Care Hospitals in Rawalpindi

**DOI:** 10.7759/cureus.21724

**Published:** 2022-01-30

**Authors:** Muhammad Sheharyar Khan, Muhammad Khurram, Shehrbano Qaiser, Najia Mahmood, Faramarz Khan, Muhammad Mujeeb Khan

**Affiliations:** 1 Internal Medicine, Rawalpindi Medical University, Islamabad, PAK; 2 Internal Medicine, Holy Family Hospital Rawalpindi, Rawalpindi, PAK; 3 Internal Medicine, Rawalpindi Medical University, Rawalpindi, PAK; 4 Internal Medicine, Fauji Foundation Hospital Rawalpindi, Islamabad, PAK; 5 Infectious Diseases, Rawalpindi Medical University, Rawalpindi, PAK

**Keywords:** covid-19, fever, alt (alanine aminotransferase), total serum bilirubin, real-time pcr

## Abstract

Background

Clinically most apparent symptoms of COVID-19 include fever and cough, which in some patients show a worsening trend but are completely non-apparent in patients who present with an asymptomatic course of the disease. The aim of this study was to identify clinical and biochemical differences among polymerase chain reaction (PCR) positive patients who are either febrile or afebrile.

Methods

This study was conducted in Rawalpindi Medical University and Allied Hospitals between September and December 2020. All patients who tested positive for reverse transcription polymerase chain reaction (RT-PCR) COVID-19 were included in the study. After evaluation of 146 patients, 100 were selected, and with a response rate of 97%, a total of 97 patients were included in the final analysis. Depending on the presence of fever, the participants were divided into two groups. Both groups were then compared for baselines vitals and laboratory investigations. Data was entered and analyzed in SPSS v23.0 (IBM Inc., Armonk, New York).

Results

Among the 97 patients, 66 (68%) of the participants were male, and 31 (32%) were females. The mean age of the study participants was 45.23±18.08 years. Fever was present in 39 (40.2%) of the participants. When compared with patients with no fever, the patients with fever had greater severity of disease (p<0.001), higher heart rate (p<0.001), decreased oxygen saturation (p<0.001). Among the laboratory investigations, the fever group had a greater tendency of having deranged alanine aminotransferase (ALT) (70.82±29.23 vs. 32.83±16.22, p=0.010), Lymphocytes (1.56±0.54 vs. 2.12±0.94, p=0.003) and serum total bilirubin (1.06±0.36 vs. 0.55±0.21, p=0.009). Based on multiple regression analysis, the presence of fever is a predictor of derangement in ALT (OR=1.034, CI=1.001-1.068 p=0.025) and total bilirubin (OR=4.38, CI=2.14-6.78, p=0.021).

Conclusion

Fever may not be present among all patients presenting with COVID-19 infection, but those who have a fever have a greater risk of having deranged liver function tests. Hence, it is important to monitor liver function tests (LFTs) in COVID-19 patients presenting with fever.

## Introduction

Coronavirus disease 2019 (COVID-19) is caused by the severe acute respiratory syndrome virus 2 (SARS-Cov-2). It manifests symptomatically in multiple ways, including fever, dry cough, dyspnea, fatigue, myalgias, and loss of appetite [1]. The symptoms vary according to the severity of the disease, ranging from anosmia to life-threatening organ failure [2]. The disease has multi-organ manifestations and has been found to be a cause of pneumonia, acute kidney injury, liver damage, diarrhea, and ischemic strokes [3].

A person infected with COVID-19 first passes through the stage of replication and then the stage of adaptation. Initially, the patient presents with flu-like illness having fever, cough, and myalgia most commonly, but as the adaptive phase begins, the patient starts to deteriorate clinically. Although the viral load reduces considerably over time, the inflammatory cascade builds up and develops into a tornado of inflammatory cytokine responses. This leads to multi-organ dysfunction [4].

Clinically most apparent symptoms of COVID-19 include fever and cough, which in some patients show an increasing trend but are completely non-apparent in patients who present with an asymptomatic course of disease [5]. The disease involves activation of an inflammatory cascade triggered by the interaction of the viral pathogen with angiotensin-converting enzyme 2 (ACE-2) receptors, eventually manifesting as fever to widespread damage to multiple organs. This may manifest as thrombotic episodes, disseminated intravascular coagulation, etc. [6]. Hence, the presence of certain symptoms poses an equal uncertainty comparable to that of the complications of the disease. The purpose of this study, henceforth, was to determine how laboratory parameters vary among patients presenting with and without fever. Clinical correlation of presenting symptoms with laboratory parameters has been minimally evaluated in the international literature and is a perspective of COVID-19 infection that needs to be examined further.

## Materials and methods

In this comparative cross-sectional study, we selected 146 patients by consecutive random sampling who presented at Rawalpindi Medical University and Allied Hospitals in Rawalpindi for the evaluation of COVID-19 infection between September 2020 and December 2020. After informed consent, a total of 146 patients were evaluated according to the selection criteria of the study.

The evaluation criteria included all those patients who tested positive by reverse transcription polymerase chain reaction (RT-PCR). The hospital protocol included all the following patients who were eligible for RT-PCR: 1) patients presenting with any two of the following symptoms: non-productive cough, fever (documented), body aches; 2) COVID-19 suspect based on radiological assessment; 3) exposure to a COVID-19 positive patient in the past seven days; and 4) patients being prepared for surgical procedures.

The selected 146 patients were asked to isolate in the hospital facility were required to undergo a second RT-PCR. Those patients who tested negative on the second RT-PCR, failed to give consent for observation in a hospital facility, and failed to give consent for laboratory investigations were excluded from the study. Patients having underlying diseases which impact the liver function tests such as chronic hepatitis, viral hepatitis, autoimmune hepatitis, drug-induced hepatitis, alcoholic liver disease, non-alcoholic fatty liver disease, liver cirrhosis, cholelithiasis, obstructive jaundice, etc. were excluded from the study after taking thorough history before the final analysis. Patients with asthma, chronic obstructive pulmonary disease (COPD), and heart failure were also excluded from the study. Patients who reported to have taken antipyretic prior to presentation were also excluded.

Eventually, a total of 100 participants were selected to be a part of the study. Among the 100 participants, there was a response rate of 97%, and all 97 participants underwent all the required laboratory investigations, clinical examination, and submission of reports. Three patients were lost due to a missing laboratory investigation due to missing reports.

**Figure 1:**
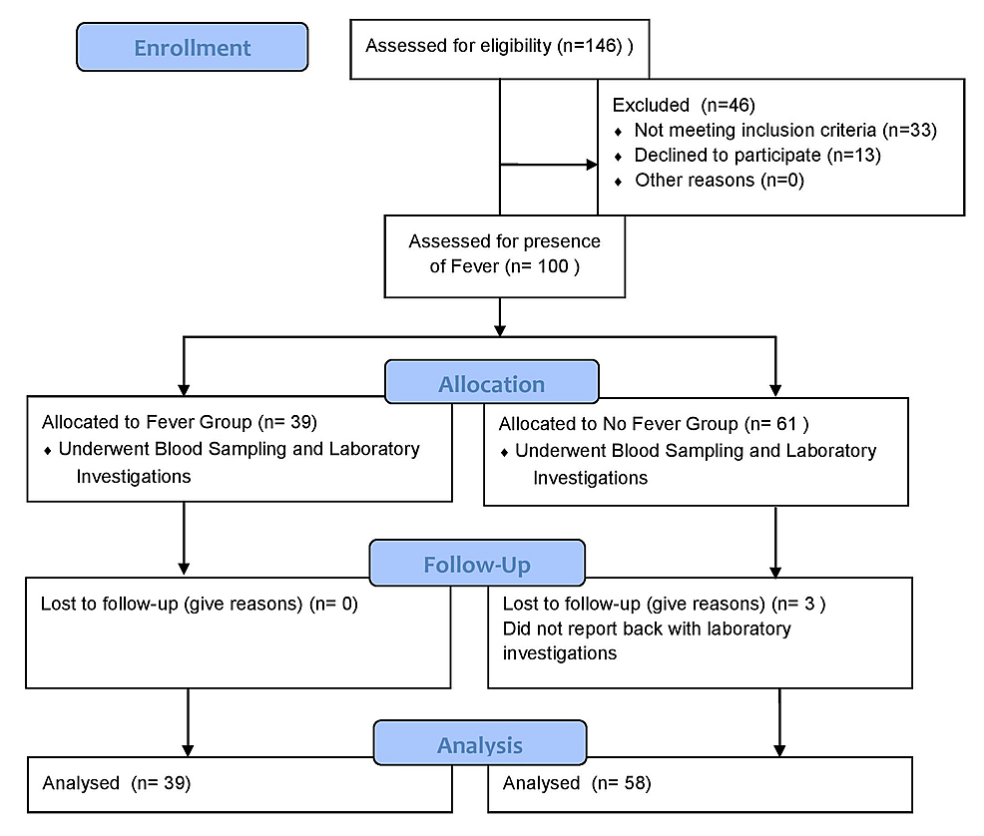
Cross-sectional study design

We divided the participants into two groups; group A: presence of a fever at the time of blood sample collection; and group B: no fever at the time of blood sample collection (Figure 1).

Fever was defined as a body temperature of greater than 98.4 F and was measured at the time of collection of blood samples. Other vitals, including heart rate, oxygen saturation, and blood pressure, were measured at the time of sample collection. All patients who tested positive on RT-PCR were isolated in a hospital facility. Laboratory samples were collected at the same time from all the patients with a constant post-prandial time. All patients were given the same diet during their stay at the hospital. Using the standard method, laboratory examination of the sample was done for complete hematological profile, liver function tests, renal function tests, and serum electrolytes.

The severity of the patients was assessed using the hospital severity assessment protocol:

• Mild: patients presenting with symptoms such as fever, cough, sore throat, headache, myalgias, loss of taste and smell, but do not have abnormal chest imaging and shortness of breath.

• Moderate: patients presenting with signs of pneumonia on chest imaging and clinical examination but have oxygen saturation (SpO2) ≥92%. Patients presenting with symptoms such as fever, cough, sore throat, headache, myalgias, loss of taste and smell, but do not have abnormal chest imaging and shortness of breath.

• Severe: patients having SpO2 <92% and a respiratory rate of >30 breaths per minute. Widespread lung infiltrates cover >50% of the lungs. Any signs of respiratory failure, septic shock, and/or multiple organ dysfunction.

Statistical analysis

Statistical analysis was conducted using SPSS version 23 (IBM Inc., Armonk, New York). All experimental values are presented as mean ± standard deviation (SD). The comparison between the two groups (group A: fever at the time of blood sample collection, group B: no fever at the time blood sample collection) was made by an independent T-test or Mann Whitney U test after testing the data for normality. The Chi-square test was used in the case of categorical variables. Laboratory parameters that were significant in univariate analysis were transformed to dichotomous variables based on optimal cut-off values using the Youden index determined by a nonparametric Kernel regression method for maximizing the summation of sensitivity and specificity. All those parameters which had a p-value less than 0.05 were added to the final regression model. Association between continuous variables (temperature and total bilirubin) was evaluated using the nonparametric Spearman's rank correlation coefficient. P-values less than 0.05 were considered statistically significant, and 95% confidence intervals were considered in all statistical tests during analysis.

## Results

Demographic variables

Out of the 97 participants in the study, 66 (68%) of the participants were male, and 31 (32%) were females. The mean age of the study participants was 45.23±18.08 years. The duration of stay in the hospital was 16.01±5.26 days for all the patients. The total number of patients presenting with fever was 39 (40.2%). Out of the 97 participants, 79 (81.4%) were clinically stable, 10 (10.3%) were clinically in moderate conditions, while eight (8.2%) were in critical condition. Table 1 shows the stratification of these variables among the two groups.

**Table 1:** Demographic variables of the patients

Variable		Fever		p-value
		Yes (n=39)	No (n=58)	
Age (years)		49.56±14.96	42.31±19.49	p=0.041
Duration of hospital stay (days)		16.19	15.90	p=0.700
Gender	Male	29 (74.4%)	37 (63.8%)	p=0.192
	Female	10 (25.6%)	21 (36.2%)	
Severity	Mild	27 (69.2%)	52 (89.7%)	p=0.033
	Moderate	6 (15.4%)	4 (6.9%)	
	Severe	6 (15.4%)	2 (3.4%)	

Vitals

Vital monitoring of the patients among the two groups showed that temperature and heart rate was higher, while oxygen saturation was lower in patients presenting with fever. These were statistically significant as shown in Table 2.

**Table 2:** Vital monitoring results of the patients SpO2 - oxygen saturation

	Fever		p-value
	Yes (n=39)	No (n=58)	
	Mean ± SD	Mean ± SD	
Temperature (Fahrenheit)	99.81 ± 1.67	98.17±0.42	p<0.001
Systolic blood pressure (mmHg)	118.72±13.80	119.30± 18.66	p=0.866
Heart rate (/min)	96.69±16.97	77.66±17.34	p=0.001
SpO2 (%)	93.69±4.66	96.98±1.55	p=0.006

Laboratory parameters

Among the two groups, the patients with fever were more likely to have raised total bilirubin and ALT while having relative lymphopenia when compared to the patients without fever. All these relations were found to be statistically significant, as shown in Table 3. Although patients with fever tend to have greater serum aspartate aminotransferase (AST) levels, these were not statistically significant.

**Table 3:** Univariate analysis of laboratory parameters among the two groups ALT - alanine aminotransferase, AST - serum aspartate aminotransferase

	Fever		p-value
	Yes (n=39)	No (n=58)	
	Mean ± SD	Mean ± SD	
Hematological profile			
Hemoglobin (g/dL)	13.70±1.69	13.45±2.53	p=0.761
Red blood cells (10^12^/mm^3^)	4.68±0.52	4.75±0.82	p=0.609
Mean corpuscular volume (fL)	88.83±11.95	87.96±8.10	p=0.581
Hematocrit (%)	42.17±4.54	41.57±8.40	p=0.712
Procalcitonin (ng/ml)	0.24±0.13	0.25±0.09	p=0.629
Mean platelet volume (fL)	8.62±-.63	8.70±1.02	p=0.468
Red cell distribution width (%)	11.09±1.17	11.91±3.57	p=0.187
Platelet distribution width (%)	9.14±1.71	8.75±1.17	p=0.448
Total leucocyte count (10^3^/uL)	7.96±3.13	8.23±2.67	p=0.459
Neutrophils (10^3^/uL)	6.11±3.13	5.83±2.24	p=0.845
Lymphocytes (10^3^/uL)	1.56±0.54	2.12±0.94	p=0.003
Monocyte (10^3^/uL)	0.26±0.15	0.36±0.22	p=0.128
Platelets (10^3^/uL)	280.00±151.91	290.00±87.68	p=0.775
Prothrombin time (seconds)	17.98±10.68	15.00±1.57	p=0.424
APTT (seconds)	30.52±9.47	35.43±7.95	p=0.232
Renal function tests			
Urea (mg/dl)	31.38±15.31	28.02±14.00	p=0.337
Creatinine (mg/dl)	1.81±4.60	0.91±0.27	p=0.069
Uric acid (mg/dl)	4.64±1.52	4.63±1.28	p=0.870
Liver function tests			
ALT (IU)	70.82±29.23	32.83±16.22	p=0.010
AST (IU)	72.39±100.47	41.47±17.01	p=0.324
Total bilirubin (mg/dl)	1.06±0.36	0.55±0.21	p=0.009
Electrolytes			
Sodium (mEq/L)	138.82±6.83	140.99±5.31	p=0.109
Potassium (mEq/L)	4.23±0.50	4.46±0.54	p=0.168
Chloride (mEq/L)	101.41±4.65	102.69±3.50	p=0.084

In the multivariable model, the significant variables were total bilirubin and ALT, as shown in Table 4. Using ALT and total bilirubin cut-off values of 42 IU and 0.6 mg/dl, respectively, multiple regression analysis showed that the presence of fever is a predictor of derangement of ALT and total bilirubin. A patient with fever is 4.368 times more likely to have a derangement in total bilirubin compared to a patient without fever.

**Table 4:** Significant variables after multivariable regression analysis of laboratory parameters

Parameters affected by fever	Odds ratio (95% CI)	p-value
Alanine transaminase (>42 IU)	1.034 (1.001, 1.068)	p=0.025
Serum total bilirubin (>0.6 mg/dl)	4.38 (2.14, 6.78)	p=0.021

Bilirubin and temperature

The spearman correlation resulted in a moderate strength of correlation between temperature and bilirubin but was highly significant (Spearman's correlation coefficient: r=0.46; p=0.001).

## Discussion

COVID-19, in its initial phase of the outbreak, was a challenge for physicians to diagnose, considering the wide range of symptomatic presentations the disease has had. However, with the progression of time, evidence has been reported in literature which has not only made the diagnosis easier than before but treatment much easier than before. Fever, in general, is produced as a physiological response for the activation of immunity in order to fight an untoward pathology within the body [7]. When considering the liver, it is proposed that the presence of ACE-2 receptors in the bile duct cells similar to their presence on type II alveolar cells and affinity to the pathogen may be responsible for the deranged liver function tests [8]. Guan et al. proved that COVID-19 is associated with microvascular steatosis and mild lobular activity in COVID-19 infection [9].

A systematic review and meta-analysis done by Grant et al. reported that fever was one of the primary symptoms associated with COVID-19, with 78% of the patients reporting having a fever once during the disease [10]. Similarly, Guan et al., however, reported that only 42.8% reported fever as a symptom when they presented with other symptoms of the disease at the time of admission [11]. Adding further, a study conducted at Rehman Medical Institute, Peshawar, reported fever in 72% of the 121 participants who tested positive. The positivity rate in this study was 14.3% [12]. Another study conducted in Karachi, Pakistan, reported fever in 83% of the patients who tested positive for COVID-19 [13]. This was, however, relatively different than our study, which reports that 40.2% of the patients had a fever. This is because our study included patients who consecutively had sampling done for RT-PCR whether they had symptoms or not, while the studies above reported fever in patients who presented with a suspicion of COVID-19 on the basis of symptoms. Asymptomatic patients such as those being prepared for surgical procedures and those getting tested for occupational purposes were included in our study while not in studies presenting a higher percentage of fever. Our study reported fever as positive when it was documented to be present at the time of sampling done for laboratory investigations. Different presentations in our study could also be due to the mutations the SAR-COV-19 have undergone over time, as our sampling process was conducted in October and November, while the studies above were in the early months of 2020. However, unlike many other studies, a study conducted in Europe presented fever as a symptom in 45.4% of the 1420 patients [14]. This study and ours add up to the literature that all patients with COVID-19 may not present with fever at all times.

Fever, like reported in other studies, has a positive correlation with the severity of the disease in the case of COVID-19. A case-control study involving a total of 142 patients reported that the occurrence of fever greater than seven days was indicative of ICU admission [15]. Fever was also proven to be an independent risk factor for severity in a study done in Zhejiang, China (OR = 3.6, 95% CI: 2.1-6.3, p<0.001) [16]. This was similar to the results presented in this study, where patients with fever had a greater chance of getting a severe disease (15.4% vs. 3.4%, p=0.031).

When discussing derangement in liver functions, our study was similar in context to other studies reported previously in literature. A retrospective study including 148 COVID-19 patients reported that 55 (37.2%) had deranged liver function tests, and patients within this group had a greater tendency of having fever (14.5% vs. 4.3%, p=0.027) [17]. Liver function test (LFT) derangement was initially reported in Wuhan in 43.4% of the patients admitted [18], and later 12 clinical studies based on multiple centers verified this finding by reporting a percentage of 14.8%-53% patients having liver function derangement along with COVID-19 infection [11-19], indicating that this association is a common occurrence. In a study by Tiang et al., mild zone 3 sinusoidal dilatation, patchy hepatic necrosis, and a mild increase in sinusoidal lymphocytes were seen in autopsies of COVID-19 patients [20]. This was further supplemented by findings of Li et al., who observed foci of hepatic necrosis adjacent to terminal hepatic veins and peri‐portal area, which are indicative of acute liver injury by a possible direct viral attack [21]. Although drug-induced injury is an unlikely mechanism reported by Fan Z et al. [16], Li et al. consider it a possible cause for exacerbation of the viral attack on the liver [21]. In addition to this, liver injury in COVID-19 infection may also be attributed to immune injury, systemic inflammatory response syndrome (SIRS), cytokine storms, ischemia, and hypoxia reperfusion injury [22]. In Pakistan, a study done in the Combined Military Hospital showed that bilirubin, ALT, AST, alkaline phosphatase, and gamma-glutamyl transferase (GGT) levels were raised in 9.6%, 15.3%, 18.2%, 15.3%, 11.5%, and 22% patients respectively, and an association of these raised levels with severity was also identified [23]. AST was marked as predictive of mortality in a study done by Lei et al. [24]. In addition to all these findings present in literature, our study presents that patients presenting with fever are more likely to have deranged liver function tests than baseline level, with emphasis on ALT (OR=1.034, CI=1.001-1.069, p=0.025) and total bilirubin (OR=4.38, CI=2.14-6.78, p=0.021) in a multivariate model. The association of fever with total bilirubin was also presented with a significant Spearman correlation. Hence, it suffices to say that a COVID-19 patient has a possibility of having deranged liver function tests, but additionally, a COVID-19 patient presenting with fever is of a greater likelihood to have hepatic involvement.

Our study has several limitations. This study had a sample size of 97 patients; for more conclusive results, we recommend studies that should include a greater number of patients. Although we examined a significant association between the presence of fever and derangement of liver function test, we could not give a definitive causality. In addition to this, our study design, by its nature, could not identify any confounding factors such as comorbidities or age, which would affect the hepatic function and the occurrence of fever at the same time. Further studies are suggested to be conducted to corroborate the causal relationship between fever and hepatic dysfunction among COVID-19 patients.

## Conclusions

This study included RT-PCR-positive patients who opted for polymerase chain reaction (PCR) testing for screening and diagnostic purposes. Many patients who were initially asymptomatic underwent the test. Hence, this study suggests that patients who are asymptomatic for COVID-19 but test positive by RT-PCR exist within our population. We cannot, as a result, predict the presence of COVID-19 infection among a population only based on symptoms.

Adding further, this study outlines the presentation of patients during the second wave of COVID infections in Pakistan. The incidence of fever during this wave is relatively less when compared to the previous waves of COVID. This reinstates the possibility that COVID-19 is evolving with time in its presentation among positive patients. 

RT-PCR-positive patients may not present with the most common symptom, fever. Moreover, those who have a fever have a greater risk of having deranged liver function tests. Hence, it is important to monitor LFTs in patients presenting with fever. Adding further, the derangement of LFTs and its coexistence with fever is not appreciated widely in literature. This aspect is not concerning for physicians who are treating COVID-19. Hence, this study provides a new perspective on the management of COVID-19 infection.
